# Two sides of the same coin? Unraveling subtle differences between human embryonic and induced pluripotent stem cells by Raman spectroscopy

**DOI:** 10.1186/s13287-017-0720-1

**Published:** 2017-11-28

**Authors:** Elvira Parrotta, Maria Teresa De Angelis, Stefania Scalise, Patrizio Candeloro, Gianluca Santamaria, Mariagrazia Paonessa, Maria Laura Coluccio, Gerardo Perozziello, Stefania De Vitis, Antonella Sgura, Elisa Coluzzi, Vincenzo Mollace, Enzo Mario Di Fabrizio, Giovanni Cuda

**Affiliations:** 1 Research Center for Advanced Biochemistry and Molecular Biology, Stem Cell Laboratory, Department of Experimental and Clinical Medicine, University “Magna Graecia” of Catanzaro, 88100 Loc., Germaneto, Catanzaro, Italy; 2 BioNEM Lab., Department of Experimental and Clinical Medicine, University “Magna Graecia” of Catanzaro, 88100 Loc., Germaneto, Catanzaro, Italy; 3 Department of Health Sciences, University “Magna Graecia” of Catanzaro, 88100 Loc., Germaneto, Catanzaro, Italy; 4 SMILEs Lab., Physical Science and Engineering Division (PSE), KAUST, 23955-6900 Thuwal, Kingdom of Saudi Arabia; 5 Department of Science, University of Rome “Roma tre”, viale G. Marconi 446, 00146 Rome, Italy

**Keywords:** Human induced pluripotent stem cells, Human embryonic stem cells, Raman imaging, Multivariate analysis

## Abstract

**Background:**

Human pluripotent stem cells, including embryonic stem cells and induced pluripotent stem cells, hold enormous promise for many biomedical applications, such as regenerative medicine, drug testing, and disease modeling. Although induced pluripotent stem cells resemble embryonic stem cells both morphologically and functionally, the extent to which these cell lines are truly equivalent, from a molecular point of view, remains controversial.

**Methods:**

Principal component analysis and K-means cluster analysis of collected Raman spectroscopy data were used for a comparative study of the biochemical fingerprint of human induced pluripotent stem cells and human embryonic stem cells. The Raman spectra analysis results were further validated by conventional biological assays.

**Results:**

Raman spectra analysis revealed that the major difference between human embryonic stem cells and induced pluripotent stem cells is due to the nucleic acid content, as shown by the strong positive peaks at 785, 1098, 1334, 1371, 1484, and 1575 cm^–1^, which is enriched in human induced pluripotent stem cells.

**Conclusions:**

Here, we report a *nonbiological* approach to discriminate human induced pluripotent stem cells from their native embryonic stem cell counterparts.

**Electronic supplementary material:**

The online version of this article (doi:10.1186/s13287-017-0720-1) contains supplementary material, which is available to authorized users.

## Background

Human pluripotent stem cells (hPSCs) include embryonic stem cells (ESCs), derived from the inner cell mass of the blastocyst [1], and induced pluripotent stem cells (iPSCs), generated from adult somatic cells by forced expression of a specific set of reprogramming factors [2, 3]. hPSCs have the ability to undergo self-renewal indefinitely while retaining the capability to differentiate into cells of all three germ layers, both in vivo and in vitro [4]. These features make hPSCs effective and advantageous cell sources for many biomedical applications, such as cell transplantation therapy [5], regenerative medicine [6], drug testing [7], and disease modeling [8]. The production and derivation of human ESCs (hESCs), however, engenders significant ethical limitations since one needs to sacrifice an embryo to harvest these cells. In addition to the ethical concerns, another crucial bottleneck for hESC-based therapy is the risk of potential allogeneic immune rejection of hESC-derived cells by recipients after cell transplantation [9]. The use of hESCs for disease modeling often involves the generation of mutant cell lines by homologous recombination for in-vitro disease recapitulation [10]. The discovery of iPSCs, as patient-specific PSCs, has overcome both of these ethical and immunological issues, making iPSCs attractive candidates to complement ESCs in research and clinical studies. Additionally, in the context of human disease modeling, patient-specific iPSCs offer advantages over human recombinant and genetically modified ESCs since hiPSCs carry the genetic and epigenetic background of the patient from which they were derived. From a morphological point of view, ESCs and iPSCs are indistinguishable; functionally, iPSCs can differentiate into cells of any of the three germ layers, like ESCs. However, despite the large similarity between ESCs and iPSCs, it is still debated whether they are molecularly equivalent [11]. Here, we use Raman spectroscopy to perform a comparative analysis of a human iPSC (hiPSC) line reprogrammed from skin fibroblasts and the commercially available hESC line H9 in order to identify specific biochemical signatures capable of discriminating between reprogrammed and native pluripotent stem cells. Raman spectroscopy is a label-free optical technique relying on inelastic light scattering, and able to provide characteristic fingerprints of biomolecules. Recently, Raman micro-spectroscopy has been employed successfully for several biochemical studies, such as lipid droplet overexpression in colorectal cancer stem cells [12], drug screening during stem cell differentiation [13], discrimination of tumor/healthy tissues [14], grading of live osteosarcoma cells [15], detection of hepatic maturation in mesenchymal stromal cells undergoing hepatic differentiation [16], screening of hESCs [17], lipidomics in living cells and tissues [18, 19], tumor-derived exosomes [20], different proteomics issues [21], as well as comparison of hESCs and hiPSCs with their differentiated derivatives [22]. Further applications of Raman spectroscopy in biochemistry and cell biology have been identified [23]. The results of Raman analysis performed on human reprogrammed and ESC lines indicate that although the overall spectral behavior is very similar for both cell lines, the hiPSC spectra still exhibit distinctive Raman features which allow for their discrimination from the native counterpart.

## Methods

### Generation and characterization of human induced pluripotent stem cells

A dermal biopsy specimen was collected after obtaining written informed consent from a healthy individual and cultured on a Petri culture dish. Fibroblasts were isolated and expanded by the outgrowth method in DMEM supplemented with 10% FBS. Fibroblasts were passaged twice and then infected for iPSC generation. Reprogramming of fibroblasts to pluripotency was performed by nonintegrating Sendai-virus-mediated transfection of the four canonical transcriptional factors (*OCT4*, *SOX2*, *KLF4*, and c-*MYC*) (CytoTune2.0 Sendai vectors; Thermo Scientific). Briefly, 3 × 10^5^ fibroblasts were infected at a multiplicity of infection (MOI) of 5, yielding different iPSC clones generated under feeder-independent conditions on Matrigel-coated dishes (BD Biosciences). Generated hiPSCs were stained for alkaline phosphatase (AP) activity (Additional file 1: Figure S1A) and subsequently picked manually for culture and propagation. Prior to performing pluripotency assays, generated hiPSCs were tested for loss of Sendai virus transgenes by RT-PCR (Additional file 1: Figure S1B). The pluripotency of generated hiPSCs and hESCs was evaluated by qRT-PCR for expression of the endogenous pluripotency genes *OCT4*, *SOX2*, *c-MYC*, *REX1*, and *NANOG* (Additional file 1: Figure S1C) and pluripotency markers Oct4 and Nanog by immunostaining (Additional file 1: Figure S1D). To further assess the pluripotency of both stem cell lines used in this study, we performed a genome-wide gene expression profile assay according to the PluriTest algorithm [24] (Additional file 1: Figure S1E). Additionally, generated hiPSCs and hESCs were tested for markers of the three germ layers, Nestin (ectoderm), Brachyury (mesoderm), and Sox17 (endoderm), on whole embryoid bodies (EBs) by immunostaining (Additional file 1: Figure S1F) and by qRT-PCR for endoderm (*SOX7*), mesoderm (*HAND1*, *ACTA2*, and *MYL2*), and ectoderm (*NESTIN* and *BMP4*) expression markers (Additional file 1: Figure S1G). hESCs (H9) were purchased from the WiCell Research Institute, and this cell line was used as a control throughout our experiments. Before performing experiments, all cell lines were tested for mycoplasma contamination.

### Cell culture

Human iPSCs and ESCs were cultured on Matrigel-coated (BD Biosciences) dishes in mTeSR1 medium (STEMCELL Technologies, Vancouver, BC, Canada). Cells were maintained at 37 °C in 5% CO_2_ in a humidified incubator. The culture medium was changed daily, and cells were passaged every 4–6 days (at 70% confluence) with Gentle Cell Dissociation reagent (STEMCELL Technologies).

### Reverse transcription PCR and quantitative real-time PCR

Reverse transcription PCR (RT-PCR) was used for Sendai viral transgene detection in infected parental fibroblasts (ipF) and loss in hiPSCs compared to their uninfected parental cells (pF). Quantitative reverse transcription PCR (qRT-PCR) was used to assess the expression of pluripotency genes as well as genes of the three germ cell layers. Total RNA was extracted using Trizol reagent (Thermo Fisher Scientific, Waltham, MA, USA) following the manufacturer’s instructions. One microgram of RNA was used for cDNA synthesis using a High-Capacity cDNA Reverse Transcription kit (Applied Biosystems). Gene expression was quantified by qRT-PCR analysis using 1 μl of the RT product and Power SYBR Green Master Mix (Applied Biosystems). qRT-PCR was performed in a StepOne Plus instrument (Applied Biosystems), and the gene expression levels were normalized to the glyceraldehyde 3-phosphate dehydrogenase (*GAPDH*) housekeeping gene. The gene expression and relative fold-change (Fc) patterns were assessed by the 2^–ΔΔCt^ method. The primers used in this work are presented in Table 1.

**Table 1:** Primers used for RT-PCR and qRT-PCR analysis

Gene	Forward primer	Reverse primer
*GAPDH*	TCCTCTGACTTCAACAGCGA	GGGTCTTACTCCTTGGAGGC
*CCNA2*	CAGCCAGACATCACTAACAGT	CCCACAAGCTGAAGTTTTCCT
*CCNB1*	GTTGGTGTCACTGCCATGTT	TGGCCAAAGTATGTTGCTCG
*CCND1*	GTCTGCGAGGAACAGAAGTG	GGATGGAGTTGTCGGTGTAG
*CCNE1*	GGAAGAGGAAGGCAAACGTG	GCAATAATCCGAGGCTTGCA
*OCT4*	GACAGGGGGAGGGGAGGAGCTAGG	CTTCCCTCCAACCAGTTGCCCCAAAC
*c-MYC*	AGAAATGTCCTGAGCAATCACC	AAGGTTGTGAGGTTGCATTTGA
*REX1*	ACCAGCACACTAGGCAAACC	TTCTGTTCACACAGGCTCCA
*KLF4*	ATAGCCTAAATGATGGTGCTTGG	AACTTTGGCTTCCTTGTTTGG
*NANOG*	TGCAAGAACTCTCCAACATCCT	ATTGCTATTCTTCGGCCAGTT
SeV*	GGATCACTAGGTGATATCGAGC	ACCAGACAAGAGTTTAAGAGATATGTATC
KOS-tg*	ATGCACCGCTACGACGTGAGCGC	ACCTTGACAATCCTGATGTGG
Klf4-tg*	TTCCTGCATGCCAGAGGAGCCC	AATGTATCGAAGGTGCTCAA
cMYC-tg*	TAACTGACTAGCAGGCTTGTCG	TCC ACATACAGTCCTGGATGATGATG
*SOX7*	TGAACGCCTTCATGGTTTG	AGCGCCTTCCACGACTTT
*HAND1*	CCAGCTACATCGCCTACCTG	CCGGTGCGTCCTTTAATCCT
*ACTA2*	GTGATCACCATCGGAAATGAA	TCATGATGCTGTTGTAGGTGGT
*MYL2*	TACGTTCGGGAAATGCTGAC	TTCTCCGTGGGTGATGATG
*NESTIN*	CAGCGTTGGAACAGAGGTTGG	TGGCACAGGTGTCTCAAGGGTAG
*BMP4*	CCTGTTGTGTGCCCACTGAAC	ATCTCAGCGGCACCCACAT

*Used for SeV genome and transgene detection in cells reprogrammed using CytoTune 2.0 Sendai vectors (Thermo Scientific)

*qRT-PCR* quantitative reverse transcription PCR, *RT-PCR* reverse transcription PCR

### Genome-wide gene expression profile

For PluriTest assays, RNA was extracted from hiPSCs and hESCs using the Stratagene Absolutely RNA kit. Total RNA (0.5 μg) was processed with an Illumina TotalPrep RNA Amplification Kit (Thermo Scientific) following the manufacturer’s instructions. The antisense RNA (aRNA) product was hybridized to the Human HT-12v4 Expression BeadChip Kit (Illumina) and run in an iSCAN system (Illumina). The raw data were uploaded to the PluriTest website (http://www.pluritest.org) and analyzed with the PluriTest algorithm [24].

### Immunofluorescence

For immunocytochemistry, hiPSCs and hESCs were fixed in 4% (vol/vol) paraformaldehyde (PFA) and subjected to immunostaining using the following primary antibodies: human Oct4 (1:400, mouse monoclonal; STEMCELL Technologies), human Nanog (1:1000, rabbit polyclonal; Abcam), human Nestin (1:1000, mouse monoclonal; STEMCELL Technologies), human Brachyury (1:20, goat polyclonal; R&D systems), and human Sox17 (1:20, goat polyclonal; R&D systems). Incubation with primary antibodies was performed overnight at 4 °C. After rinsing with Dulbecco’s phosphate-buffered saline (DPBS), goat anti-mouse Alexa-Fluor-647, donkey anti-Goat Alexa-Fluor-594, and goat anti-rabbit Alexa-Fluor-488-conjugated secondary antibodies (all from Thermo Scientific) were added, and cells were incubated for 1 hour at 37 °C. Nuclei were counterstained with 4′-6-diamidino-2-phenylindole (DAPI). Slides were mounted with Fluorescent mounting medium (Dako Cytomation), and microscopy was performed using imaging systems (DMi8), filter cubes, and software from Leica microsystems.

### DNA and RNA analyses for nucleic acid quantification and gel electrophoresis

Genomic DNA (gDNA) from hiPSCs and hESCs was extracted using a GenElute Mammalian Genomic DNA Miniprep kit (Sigma Aldrich, Saint Louis, MO, USA), while total RNA was extracted using an Absolutely RNA Miniprep kit (Agilent Technologies). Prior to DNA/RNA extraction, hiPSCs and hESCs were counted, and 4 × 10^5^ cells were processed for nucleic acid purification. DNA and RNA samples were eluted in an equal volume of elution buffer, and 1 μl of each DNA/RNA sample was used for quantification by a NanoDrop spectrophotometer (Thermo Fisher Scientific); 0.5 μg of each RNA and DNA sample were loaded onto 1% agarose gels for electrophoresis and mass quantification. Nucleic acid purification and agarose gel electrophoresis were performed in biological triplicate for each cell line tested.

### Mitotracker staining

For mitochondrial labeling and activity, hiPSCs and hESCs were incubated for 30 minutes at 37 °C with 100 nM MitoTracker Green FM (Thermo Fisher Scientific) diluted in growth medium (mTeSR1; STEMCELL Technologies). Fluorescence was measured with a Leica imaging system (DMi8), and the fluorescence intensity (magnification × 20) was analyzed using Leica LAS-X software. The results are presented as the mean ± standard deviation (SD) of three independent experiments.

### Cell proliferation assay by CFSE

Cell proliferation assays of hiPSCs and hESCs were evaluated by the 5,6-carboxyfluorescein diacetate succinimidyl ester (CFSE) method. Briefly, 5 × 10^5^ cells were labeled with 8 μM CellTrace CFSE (cell proliferation kit; Thermo Fisher Scientific) in mTeSR1 medium for 10 minutes at 37 °C. Labeling was quenched by adding cold PBS with 0.1% bovine serum albumin (BSA) to cells, followed by a 5-minute incubation on ice. Two hours later (T0) and after 4 days (T4) of culture in mTeSR1 medium, cells were harvested for CFSE fluorescence evaluation by flow cytometric analysis (BD LSRFortessa x-20). Cell proliferation was calculated by measuring the decrease in label intensity in successive daughter cell generations [25]. The proliferation index and cell populations of parental or successive generations were calculated with Modfit LT Version 3.2 software.

### Propidium iodide staining for cell cycle analysis

Analysis of the cell cycle status was performed by flow cytometry on cells labeled with propidium iodide (PI), a fluorescent intercalating agent that is used to assess the DNA content during the cell cycle. For this assay, hiPSCs and hESCs were treated by Accutase for single cell dissociation, and 5 × 10^5^ cells were harvested in PBS and alcohol-fixed with 70% cold ethanol at 4 °C for 30 minutes. After fixation, cells were washed three times with cold PBS, spun, and treated with PBS containing 0.1% Triton, 5 μg/ml PI, and 5 μg/ml ribonuclease for 1 hour in the dark. PI-stained cells were then analyzed by flow cytometry (BD LSRFortessa x-20) for proliferation and cell cycle distribution estimation.

### Karyotyping

Karyotype analysis of hiPSCs and hESCs was performed by multiplex-fluorescence in-situ hybridization (M-Fish). Cells were treated with KaryoMAX Colcemid solution (Thermo Fisher Scientific) and processed with standard methods. Briefly, fixed cells dropped onto glass slides were hybridized with the 24XCyte Human Multicolor FISH Probe Kit (MetaSystems, Altlussheim, Germany), following the manufacturer’s instructions. Slides were denatured in 0.07 N NaOH and then rinsed in graded ethanol. Meanwhile, the probe mix was denatured in a MJ mini personal thermal cycler (Bio-Rad Laboratories, Hercules, CA, USA) with the following program: 5 minutes at 75 °C, 30 seconds at 10 °C, and 30 minutes at 37 °C. Samples were then hybridized in a humidified chamber at 37 °C for 48 hours, followed by one wash in saline–sodium citrate (SSC) buffer for 5 minutes at 75 °C and counterstaining with DAPI. Finally, metaphases were visualized and captured using an Axio-Imager Z2 microscope. Karyotyping analysis was performed by means of ISIS software. To determine the karyotype of the hiPSCs and hESCs, 50 metaphase spreads were analyzed.

### Embryoid body formation

For EB formation, hiPSCs and hESCs were dissociated into single cells by Accutase (Thermo Fisher Scientific) and cultured on an ultralow attachment plate (Corning) with mTeSR1 medium supplemented with 10 μM Rho-kinase inhibitor Y-27632 (Selleckchem) for 3 days to enable cell aggregation. After 3 days, the medium was switched to DMEM/F12 containing a 20% knockout serum replacement (KSR), 2 mM l-glutamine, 1 × 10^−4^ M nonessential amino acids, 1 × 10^−4^ M 2-mercaptoethanol, and 0.5% penicillin and streptomycin (all from Thermo Fisher Scientific). The medium was changed every other day until day 8 [1]. After 8 days in culture as floating EBs, cell aggregates were transferred onto 0.1% gelatin-coated plates (Sigma-Aldrich) and cultured in the same medium for an additional 8 days before collecting the EBs for immunofluorescence and qRT-PCR analyses.

### hPSC culture for Raman spectroscopy measurements

For Raman spectroscopy, hiPSCs and hESCs (all at passage P40) were dissociated into single cells by Accutase (Thermo Fisher Scientific), and 4 × 10^5^ cells per cell line were seeded on CaF_2_ slides because of its negligible Raman signal for 24 hours to allow the cells to adhere to the CaF_2_ surface in mTeSR1 medium. Prior to Raman measurements, cells were fixed with 3.7% formaldehyde (Sigma-Aldrich) for 15 minutes at room temperature. After incubation in a fixative solution, cells were rinsed with DPBS and kept in distilled water for analysis to reduce the background interference derived from the culture medium.

### Raman mapping and spectra preprocessing

Raman imaging was performed with an Alpha-300R microscope from Witec GmbH (Ulm, Germany) equipped with a 532-nm laser source in a backscattering configuration. The total laser power applied to the sample was set to 10 mW to avoid cell photodamage, and light was focused on the sample through a 100×/0.9 NA objective. A 600 lines/mm grating was used for frequency analysis of the backscattered light, with a spectral resolution of approximately 3.0 cm^–1^. For each measured cell, Raman maps were recorded using a raster scan with a step size of 400 nm, which is close to the optical resolution of the system (≈360 nm) as calculated with the Rayleigh criteria. For each pixel, we used a typical integration time of 2.0 seconds, with a spectral window ranging from 400 to 3100 cm^–1^. The Raman shift was previously calibrated by measuring a silicon sample and using the sharp Si-peak at 520 cm^–1^ as a reference. After Raman measurements, the spectra were first divided into two spectral regions: one ranging from 400 to 1800 cm^–1^, which is the well-known fingerprint region; and a second region ranging from 2600 to 3100 cm^–1^, in which the CH_2_ and CH_3_ stretching vibrations were located. The spectra collected from the surrounding area of the cells were used as background spectra and subtracted from the cell signals. Finally, all of the spectra of one map were normalized to the maximum total spectral area recorded for that specific cell, allowing a comparison of Raman maps recorded from different cells at different times.

### Multivariate analysis

Principal component analysis (PCA) and K-means cluster analysis (KCA) were performed on the collected datasets. To compare the results of multivariate analysis between different maps, the spectra from all the probed cells were processed altogether as one single collection, and the computed principal components (PCs) were exactly the same for all maps. The first six PCs, which comprised more than 98% of the total variance, were used to perform KCA, imposing six clusters to be addressed inside the cells (plus one cluster collecting the empty areas outside the cells). Subsequently, pseudo-color images were generated to represent the multivariate results. A specific color was assigned to each cluster and the cluster’s spatial distribution was mapped in the *xy* space. A custom-developed software package, Raman Tool Set, freely available online (http://ramantoolset.sourceforge.net) [26], was used to perform all of the spectra preprocessing steps and multivariate analysis.

### Statistical analysis

All experiments were performed at least three times, each in biological replicates. Data were analyzed using GraphPad Prism 6 software, and statistical analysis was performed by Student’s *t* test. All values are expressed as the mean ± standard error of the mean (SEM) in all figure panels in which error bars are shown, and differences with *p* < 0.05, *p* < 0.01, and *p* < 0.001 were considered statistically significant.

## Results

**Fig. 1:**
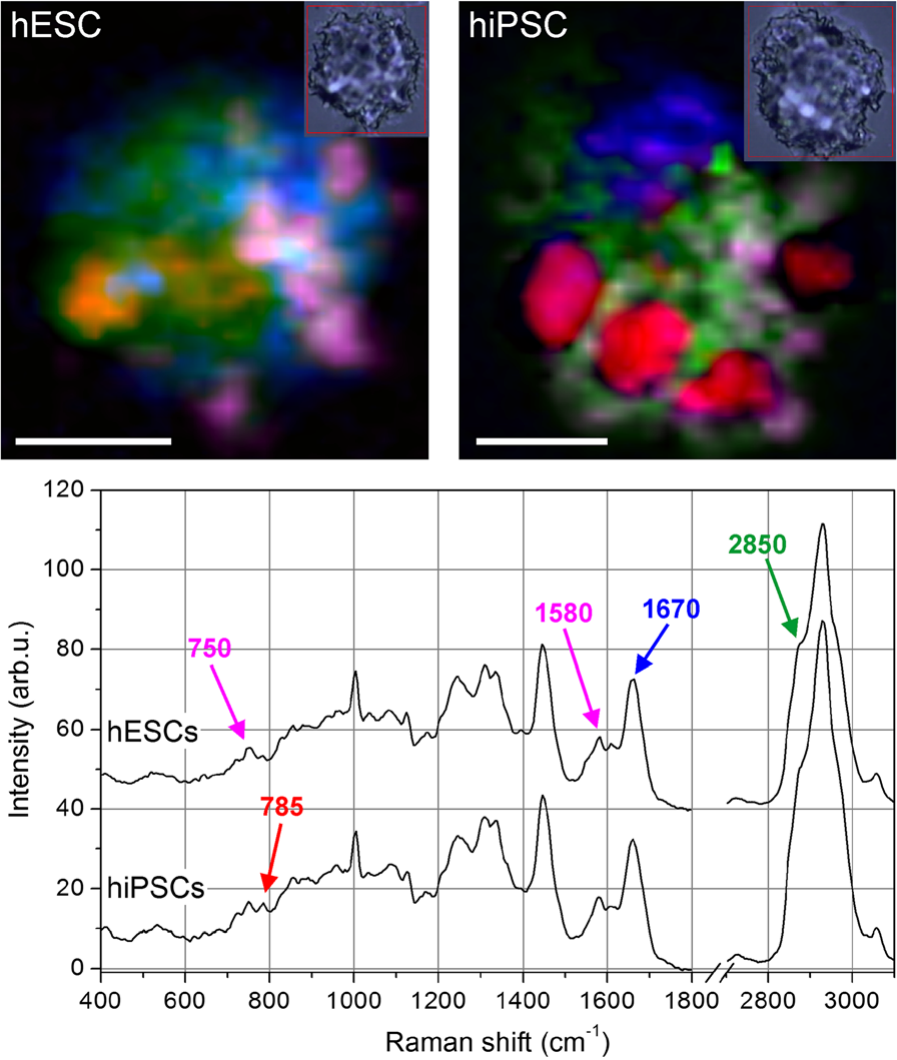
Raman imaging of typical human ESCs and iPSCs. Color-reconstituted Raman images of human embryonic stem cells (hESCs, upper left panel) and human induced pluripotent stem cells (hiPSCs) (upper right panel). White scale bar = 5 μm. Small insets show corresponding bright-field images recorded after Raman scanning. Raman peak at 785 cm^–1^ (DNA/RNA bases) mapped in red, 1670 cm^–1^ (proteins) in blue, 2850 cm^–1^ (lipids) in green, and a combination of 748 and 1585 cm^–1^ (cytochrome C) in magenta. hiPSCs exhibit a much higher level of DNA/RNA bases in well-defined regions of the cell. Curves in the lower panel are average spectra of hESCs (top curve) and hiPSCs (bottom curve), where the peaks used for the color-reconstituted images are indicated with the corresponding color

**Fig. 2:**
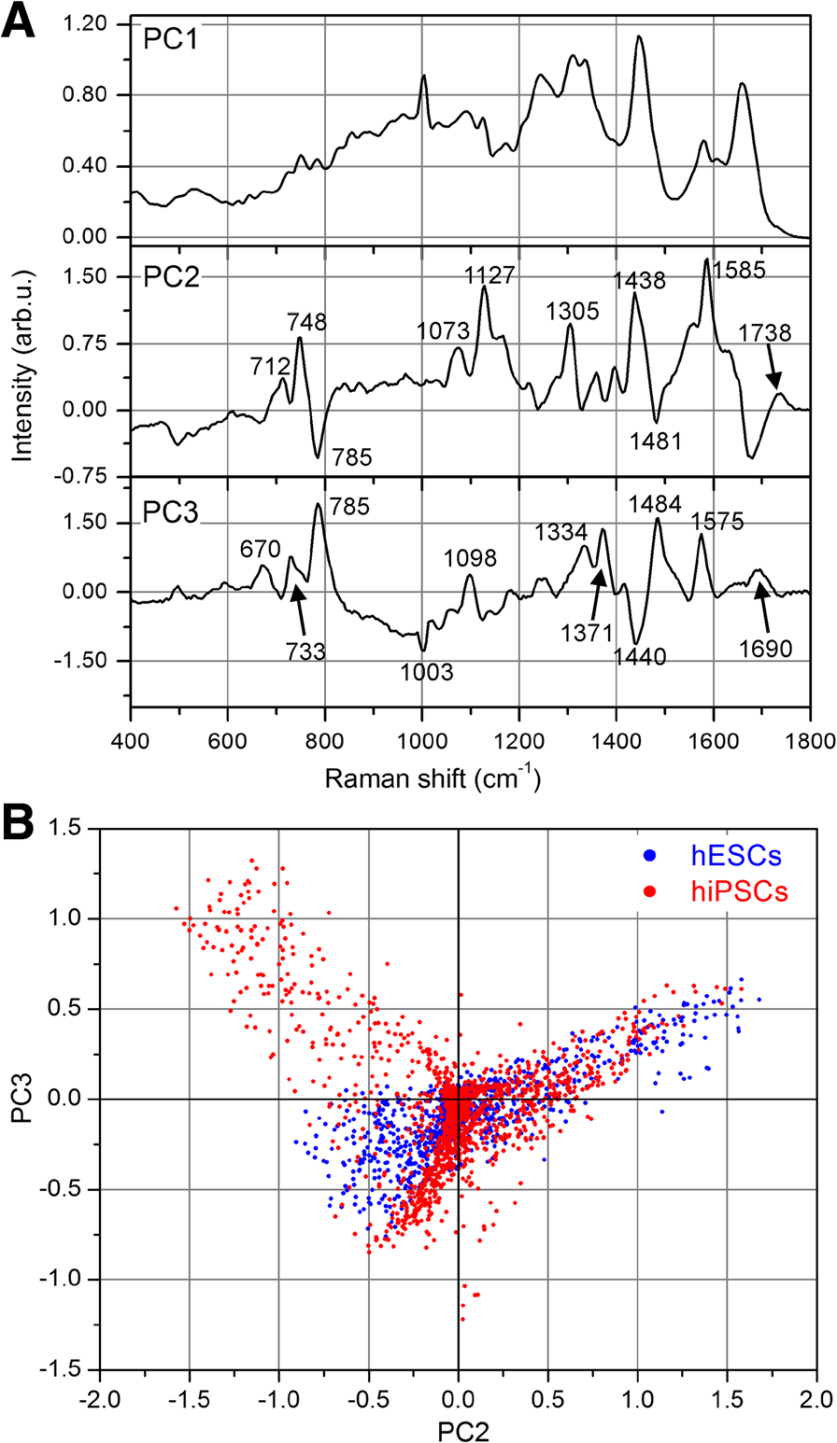
Principal component (PC) curves as biochemical indicators. **a** Loading curves of the first three PCs calculated by PCA. Since PCA is performed on the overall dataset of all probed cells, the computed PCs are the same throughout all of the measured spectra, and their characteristic bands indicate sensitive biochemical features (see text for details concerning peak assignment). While the PC1 curve only resembles the global average spectrum, the PC2 and PC3 curves account for the significant biochemical differences between the different cells, as well as different regions of the same cell. Scatter plot of PC3 vs PC2 scores for hESCs (blue dots) and hiPSCs (red dots) from Fig. 1. Each dot corresponds to one spectrum (pixel) of Raman mapping. Blue and red dots closely overlap, except for the top-left part of the graph which corresponds to positive PC3 and negative PC2 scores (**b**). Loading curves of (**a**) support that this PC3–PC2 region can be assigned to DNA/RNA bases, the typical frequencies of which are exhibited as positive bands in the PC3 curve (mainly 785, 1098, 1484, and 1575 cm^–1^), and as a sharp negative band (785 cm^–1^) for the PC2 curve. hESC human embryonic stem cell, hiPSC human induced pluripotent stem cell

**Fig. 3:**
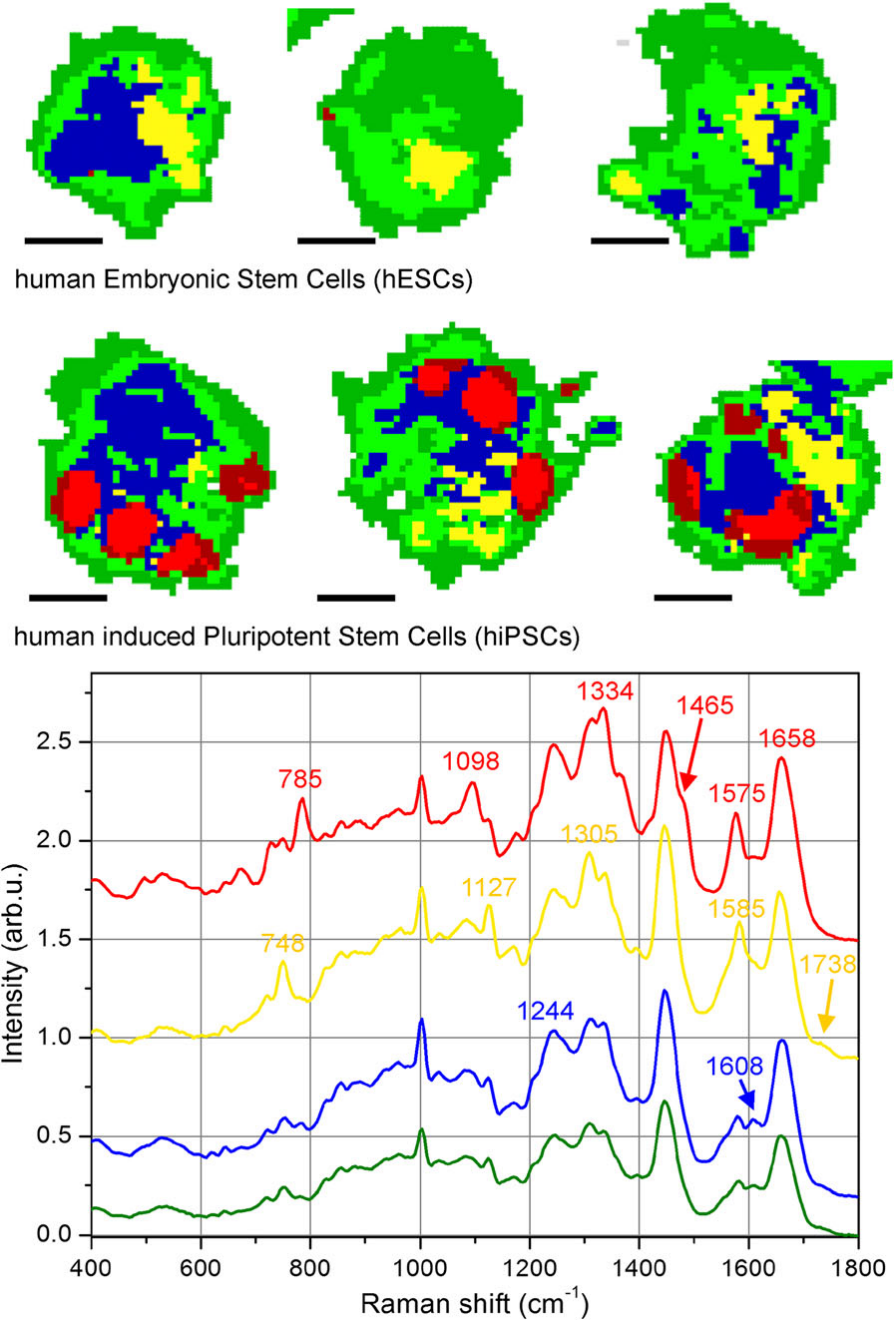
Semiquantitative comparison of Raman images by cluster analysis. KCA performed on the PCA results for Raman assignment of different cellular regions. Top row reports results for three typical hESCs, while second row reports results for three typical hiPSCs (scale bar = 5 μm). For the KCA calculation, six clusters were imposed within the cells (see text for further details), and the red cluster is only evident in hiPSCs (upper panel). Lower graph shows average Raman spectra of each cluster, where the curves have the same color as the corresponding cluster. The red curve exhibits all of the major peaks ascribed to DNA/RNA bases, and consequently the red regions inside the cells are assigned to DNA/RNA compartments. The absence of red clusters inside hESCs does not mean that DNA/RNA bases are missing therein, only that their expression is much lower than the DNA/RNA abundance in the red regions of hiPSCs

Figure 1 shows two typical cells probed by Raman micro-spectroscopy, one hESC and one hiPSC. The smaller insets display bright-field pictures of the cells recorded by the optical microscope equipping the Raman spectrometer, while the larger pictures show the corresponding color-reconstituted Raman images. The Raman intensities for peaks at 1670 cm^–1^ (proteins), 2850 cm^–1^ (lipids), 785 cm^–1^ (nucleic acids), and the combination of 748 cm^–1^ with 1585 cm^–1^ (cytochrome C) are mapped in blue, green, red, and magenta, respectively. The curves reported in the bottom panel represent the overall averaged spectra of the two cells, and the Raman bands used for the pseudo-color pictures are clearly indicated. It is immediately evident that the hiPSC exhibits a more intense Raman signal corresponding to nucleic acids and that this overexpression is localized in well-defined regions inside the cell. To achieve a semiquantitative comparison between hESCs and hiPSCs, multivariate analysis (PCA followed by KCA) was performed over the whole recorded dataset including all of the mapped cells. The loading curves of the first three principal components (PCs) are shown in Fig. 2a. Even if PCA is performed on the relative spectra (i.e., after subtraction of the overall mean spectrum), the PC1 loading curve (upper panel of Fig. 2a) correctly resembles the average cell spectrum because it discriminates between the inner and outer regions of the cells in the Raman maps. By contrast, the following principal components, PC2 and PC3, provide much more information regarding the cell biochemical composition. In the PC2 loading curve (middle panel of Fig. 2a), the positive peaks located at 748, 1127, and 1585 cm^–1^ are due to cytochrome C (cyt c) vibrations [27, 28]; their sharpness and relatively high intensities are due to the resonant Raman scattering that cyt c undergoes under 532-nm laser light [29]. The peak at 1305 cm^–1^ could be assigned both to the Amide III vibration [30, 31] as well as to vibrations due to fatty acids [19, 31]. The signature at 1438 cm^–1^ likely arises in PC2 from the asymmetric behavior of the C–H Raman band at 1440–1450 cm^–1^ across all spectra: when this latter band is closer to 1440 cm^–1^, the corresponding C–H vibration is typical of lipids (vice versa for proteins). The 1073 cm^–1^ Raman feature is typical of a C–C stretch in gauche lipids [19]. Consequently, the PC2 loads are dominated by the resonant scattering of cyt c, with a smaller contribution coming from lipids and disordered lipids (the gauche phase is typical of liquid-state lipids). The PC3 curve instead exhibits strong positive peaks at 785, 1098, 1334, 1371, 1484, and 1575 cm^–1^, all of which can be ascribed to DNA (and/or RNA) molecules as reported previously [32–34]. The Raman peaks at 785 and 1484 cm^–1^ are due, respectively, to pyrimidine vibrations [32, 33] and purine stretches [34] and, consequently, are ascribed to Cytosine and Thymine bases and Guanine and Adenine bases, respectively. The 1334 and 1371 cm^–1^ Raman peaks are caused by aromatic vibrations of DNA bases, while the 1575 cm^–1^ peak is due to Adenine and Guanine only. Finally, the smaller 1098 cm^–1^ band originates from the backbone vibration of DNA (PO^2–^ group). Even using the scores of the first six PCs as inputs for KCA, we report a detailed comment on the Raman bands only for the first three PCs since they account for almost 98% of the overall signal variance, while PC4, PC5, and PC6 together account only for 0.5% of the overall signal variance. Furthermore, no noise reduction based on the first six PCs was performed on the data, and consequently KCA assigns spectra to different clusters according to the Euclidean distances in the PC1–PC6 space, but without removing potential features that come from other PCs. KCA is performed on all cells together, imposing a total number of seven clusters. One cluster always collects spectra outside of cells, which are easily discriminated by KCA because of their very small intensities. Consequently, six clusters of spectra are distinguished inside cells. Tests performed with a higher number of clusters resulted in redundant groups without any further information. By contrast, a lower number of clusters produced excessively large variances within each cluster, indicating that a relevant number of spectra are “forcibly” assigned to the groups. The spatial distribution of the six clusters is shown in Fig. 3. Except for the red clusters, the other colors have no direct correspondence with the colors shown in Fig. 1 since they show, respectively, the results of multivariate (Fig. 3) and univariate (Fig. 1) analyses. It is evident that the red and dark red clusters are only present in the hiPSC lines, while hESCs exhibit very few or no red pixels. Other color labels, such as blue, yellow, and green (dark and light), are detected in both cell lines. The different expression levels of the blue and yellow areas in the measured hESCs are likely due to the different cell cycle phases of each single cell before fixation and Raman analysis. Compared to hESCs, hiPSCs show a more uniform behavior. The bottom panel of Fig. 3 is a graphical representation of the average spectra of each cluster, with the color of the curves corresponding to the colors of the clusters. Dark green and dark red curves are not reported because their general behaviors and major peaks are exactly the same as those of the light green and light red curves, but with a smaller intensity. This panel also reports the Raman frequencies of those peaks having different intensities, from the top curve (red) to the bottom curve (green). The red spectrum is characterized by peaks at 785, 1098, 1334, and 1575 cm^–1^, which are DNA/RNA-base-related vibrations (see the earlier peak assignment discussion for the PC2 and PC3 curves). Moreover, in the red curve, two protein markers show differences from the other spectra: the shoulder at 1465 cm^–1^, on the right side of the C–H band at 1440–1450 cm^–1^, is typical of a higher protein content [35]; and the Amide I band at 1650–1680 cm^–1^ is centered at 1658 cm^–1^, at lower frequencies than the other spectra, indicating a higher expression of alpha-helix structures [36]. No changes are detected in the behavior of the peak at 1003 cm^–1^, assigned to the breathing mode of phenylalanine, and the band at 1244 cm^–1^, assigned to the Amide III vibration [37]. In the following yellow curve, peaks at 748, 1127, and 1585 cm^–1^ are highlighted and generated from cyt c molecules, along with a band at 1305 cm^–1^, likely derived from lipids [18, 19]. Moreover, a small shoulder at 1738 cm^–1^, compared to the other average spectra, is observed. This latter signal is also typical of lipids; that is, the C = O stretch in ester groups [18]. In the average spectrum of the blue cluster, characteristic signatures are found at 1244 cm^–1^, with a higher intensity ratio *I*_1244_/*I*_1305_ compared to the other clusters spectra, and at 1608 cm^–1^, with a small band largely missing in the other curves. As already stated, the former signature (1244 cm^–1^) is a characteristic vibration of the Amide III band, while the latter signature (1608 cm^–1^) is the overlapping of ring-breathing modes of phenylalanine and tyrosine with a C = C vibration mode in proteins [38], which ascribes these signals to proteins. Finally, the green curve has an overall intensity smaller than those of the others, and the profile of the curve, as well as the relative ratios of the main peaks, closely resemble the PC1 loading curve (which is an overall average, as discussed earlier). The significant decrease in intensity is due to the scattering volume: since the green areas correspond to thinner regions of cells (see optical images in Fig. 1), the laser spot along the *z* axis could be partially outside the cell, thus leading to a smaller amount of scattering molecules. The green areas correspond to outer regions of the cells (such as external membranes) and/or to the most adherent (thin) areas, where organelles are unexpected. It is worth noting that the lack of red clusters in hESCs in Fig. 3 does not mean that DNA/RNA bases are missing in these cells. In fact, Fig. 1 shows DNA/RNA red regions for both cell lines. But the DNA/RNA Raman intensity is so high in the red cluster regions of hiPSCs that it overcomes the other Raman signals, while this is not occurring in the hESCs. A more quantitative evaluation was achieved from KCA by computing the percentage area of each cluster. Comparisons between the percentage values of hESCs and hiPSCs show that, along with a strong variation in nucleic acid content (comprised of the red cluster), no significant changes were observed in the cyt c intracellular distribution (composed of the yellow cluster) between the two cell lines. In sum, cluster analysis of the Raman images conclusively demonstrates that hESCs have much lower nucleic acid content than hiPSCs.

### Biological validation of the Raman results

**Fig. 4:**
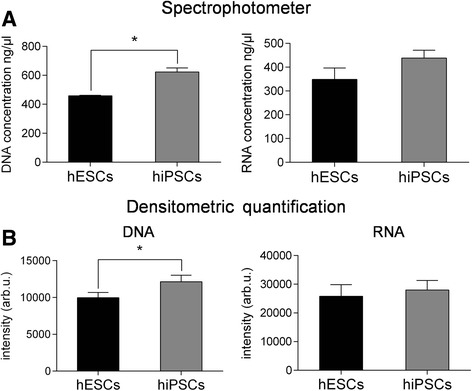
Quantification of nucleic acid levels. DNA and RNA extracted from both pluripotent stem cell lines were quantified with a NanoDrop 2000 UV-Vis spectrophotometer (**a**) and with ethidium bromide staining on agarose gel electrophoresis (**b**). Error bars indicate mean ± SEM. Statistical comparison between hiPSCs and hESCs by paired Student’s *t* test (*p < 0.05). hESC human embryonic stem cell, hiPSC human induced pluripotent stem cell

**Fig. 5:**
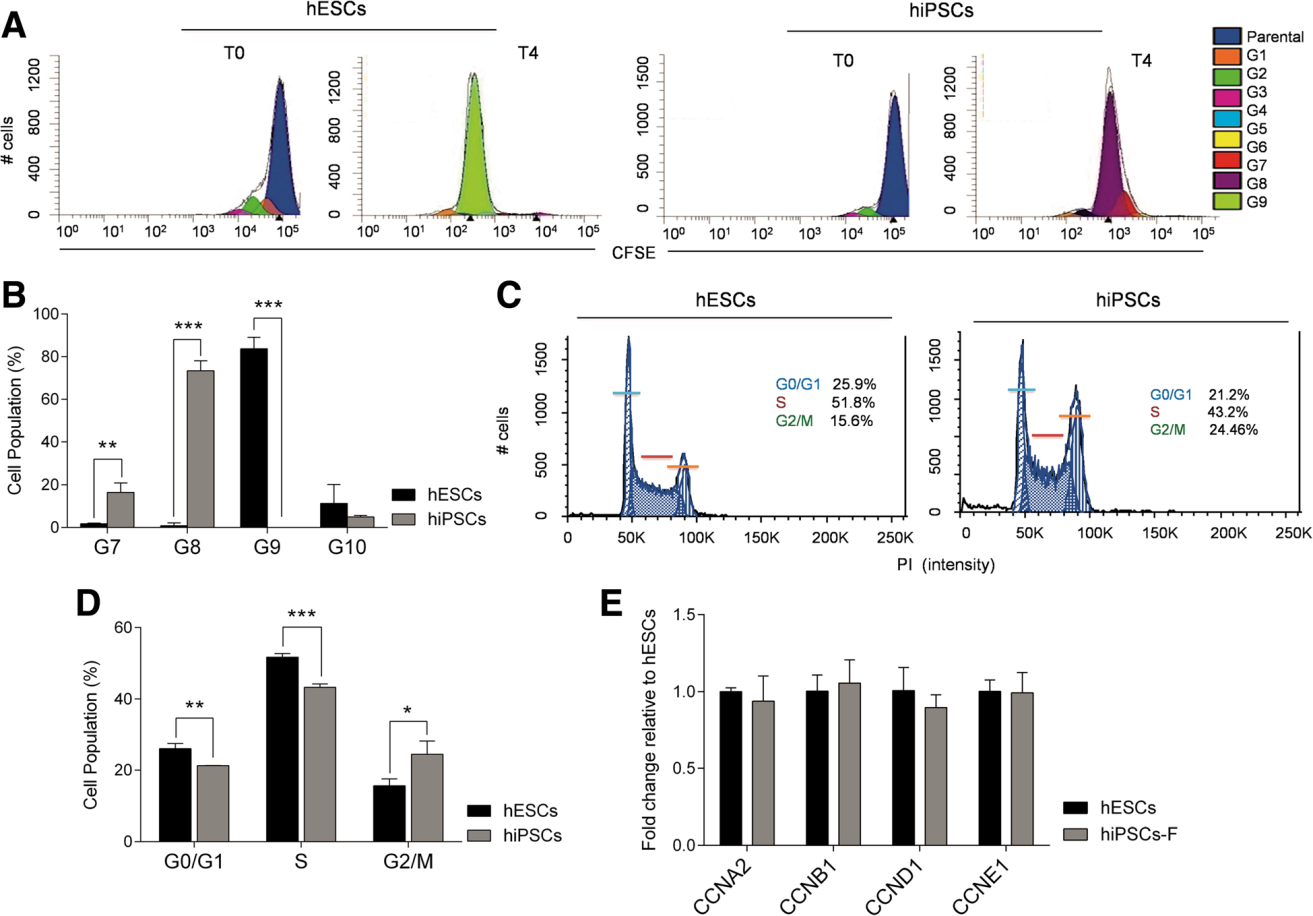
Cell cycle and proliferation rate analysis. **a** Flow cytometric analysis of hESCs and hiPSCs stained with CFSE and cultured for 2 h (T0) and 4 days (T4) after staining. **b** Proliferation rate of hiPSCs quantified and compared to that of hESCs. Quantitative data expressed as mean ± SD of three independent experiments. Statistical comparison for each generation by paired Student’s *t* test (**p < 0.01, ***p < 0.001). **c** Cell cycle progression analysis of hiPSCs and hESCs. Cells were stained with propidium iodide (PI) and analyzed by fluorescence-activated cell sorting. Data shown as mean ± SD from three independent experiments. **d** Statistical comparison between hiPSCs and hESCs for each phase of the cell cycle by paired Student’s *t* test (*p < 0.05, **p < 0.01, ***p < 0.001). **e** Quantitative real-time PCR (qRT-PCR) analysis of the cell-cycle-associated proteins *CCNA2*, *CCNB1*, *CCND1*, and *CCNE1* in hESCs and hiPSCs. All expression values normalized to *GAPDH* and relative to hESCs. Data represent the mean ± SD from three independent experiments. CFSE 5,6-carboxyfluorescein diacetate succinimidyl ester, hESC human embryonic stem cell, hiPSC human induced pluripotent stem cell

**Fig. 6:**
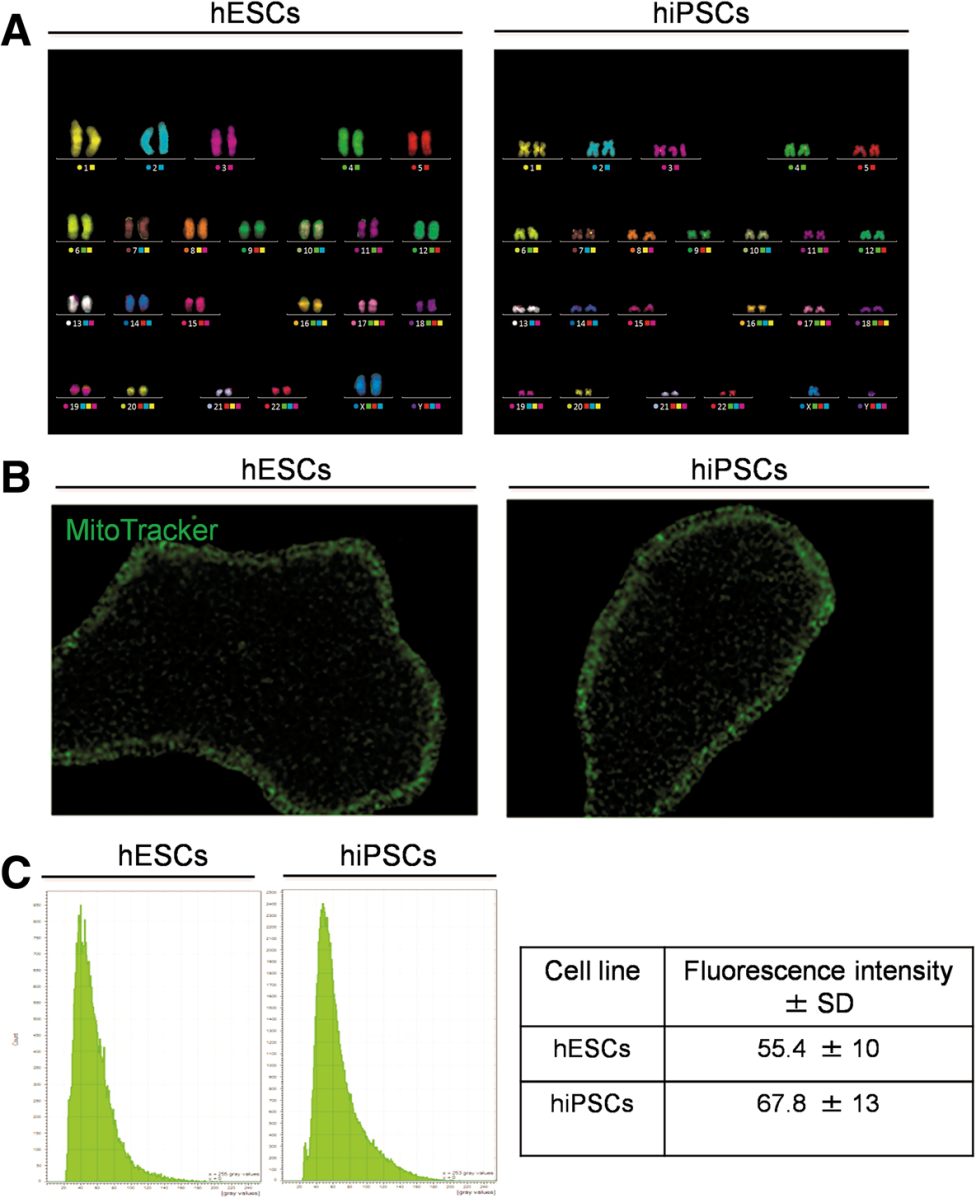
Karyotype analysis and fluorescence-based quantification of mitochondria. **a** Representative image and karyotype of an M-Fish stained hESC (left) and an M-Fish stained hiPSC (right), confirming that both cell lines have normal karyotypes. **b** Mitochondrial staining using the MitoTracker Green FM of hESC and hiPSCs. Magnification × 20. **c** Representative graphs of mean fluorescence intensity values in a single cell colony and average intensity ± SD in two cell lines. hESC human embryonic stem cell, hiPSC human induced pluripotent stem cell

The results of the Raman spectra analysis revealed a specific fingerprint for hiPSCs with respect to hESCs. In particular, major differences were found in the nucleic acid content (DNA and/or RNA), as demonstrated by the spectral features at 785, 1098, 1334, and 1575 cm^–1^ (all DNA/RNA-base-related peaks), which are significantly higher in hiPSCs compared to their hESC counterparts. Based on the Raman spectroscopy results, we biologically validated these findings using different conventional approaches. First, we verified that the nucleic acid content was indeed higher in hiPSCs compared to hESCs. This validation step was performed by nucleic acid extraction from 4 × 10^5^ cells per group, as described in Methods. After purification, 1 μl of DNA and RNA samples was used for spectrophotometric analysis by a NanoDrop instrument. As shown in Fig. 4a (left graph), the cumulative levels of DNA were significantly higher in hiPSCs compared to hESCs. Analogous results, albeit somewhat less striking, were obtained by measuring the RNA content (Fig. 4a, right graph). To confirm these findings by an independent approach, we loaded 0.5 μg of total DNA and 0.5 μg of total RNA onto a 1% agarose gel for mass quantification. As shown in Fig. 4b, the same trend was observed. Altogether, these results suggest that Raman spectroscopy represents an extremely accurate and sensitive method for detecting even subtle quantitative and qualitative differences among highly homogeneous cell lines. Differences in the DNA level may reflect a different proliferation rate that, in our case, is not age related since the hiPSCs and hESCs used in this study were all at the same passage (P40). Since the DNA level undergoes significant variations during the cell cycle, we further investigated the Raman findings by performing flow cytometry assays based on the dilution of CSFE fluorescence, which relies on intensity-halving at each cell division. In our experimental setting, we measured the CFSE intensity at time zero (T0) to define the first generation (G1, 5 × 10^5^) and reanalyzed the cells at day 4 (T4) to monitor the rounds of cell cycles in subsequent generations. To characterize the CFSE distribution, flow cytometry data were analyzed by Modfit LT Version 3.2 software using a statistical tool known as the proliferation index, which corresponds to the average number of cell divisions that a cell in the original population has undergone [39]. Graphs and proliferation indexes relative to the cell cycle events in each of the cell lines analyzed are shown in Fig. 5a, b. Intriguingly, hESCs occupied a higher position in terms of the proliferation rate, with 83% of cells in G9 after 4 days of culture, while the majority of hiPSCs (73%) were still in G8 at T4. To gain further insight regarding the DNA level and quantify the percentage of hiPSCs and hESCs within the G1, S, and G2/M phases, we performed a cell-cycle kinetic assay by PI staining, followed by flow cytometry. As shown in Fig. 5c, no significant differences in cell cycle progression were detected. More specifically, the cell cycle profiles of the hESC populations showed that 25.9% of cells were in G0/G1, 15.6% were in G2/M, and 51.8% were in the S phase, while the cell cycle profile of hiPSCs showed 21.2% of cells in G0/G1, 24.46% in G2/M, and 43.2% in the S phase. A graph relative to the cell-cycle kinetics is shown in Fig. 5d. Furthermore, we also determined the expression levels of proliferation-associated proteins, such as cyclin A (*CCNA2*), cyclin B1 (*CCNB1*), cyclin D (*CCND1*), and cyclin E (*CCNE1*), by qRT-PCR analysis (Fig. 5e). The cyclin profiling results followed the same trend in all of the tested pluripotent stem cells, with negligible differences between hiPSCs and hESCs. We further deepened our analysis by performing a karyotype study to exclude chromosomal aberrations in hiPSCs, which are often reported to be a consequence of cellular reprogramming [40]. M-Fish analysis of 50 metaphases ruled out any such abnormalities (Fig. 6a). In addition to the differences in the DNA/RNA level, Raman spectra analysis also highlighted clear peaks at 748, 1127, and 1585 cm^–1^, all of which are cytochrome C-related and do not exhibit any appreciable cyt c difference between the cell lines. To validate these results, we performed MitoTracker staining to selectively label mitochondria, the inner membrane of which is associated with the cytochrome C complex. As shown in Fig. 6b, c, we could not detect any significant difference in the mitochondria staining intensity between hESCs and hiPSCs, thus supporting the Raman analysis results.

## Discussion

In the present study, Raman spectroscopy was used to perform a comparative analysis of hESCs and hiPSCs. The experimental results demonstrate that even though the pluripotent stem cell lines analyzed are largely equivalent, small but significant differences can be appreciated. Raman spectroscopy is well suited for detecting subtle differences in specific biochemical groups. In our case, a multivariate analysis of peak intensities highlights a dissimilarity between the two pluripotent stem cell lines in terms of the nucleic acid levels, with a higher amount detected in hiPSCs compared to hESCs. At the present date, we do not have a clear mechanistic explanation for these differences; with regards to nucleic acids, we hypothesize that the different epigenetic backgrounds between hiPSCs and hESCs might play at least in part a significant role. ESCs are known to be transcriptionally hyperactive, undergoing major silencing during differentiation [41]; moreover, differences in chromatin dynamics are likely to occur in iPSCs vs ESCs and have been reported by several groups [41, 42]. Tan et al. [22] have shown, using a similar approach, that hiPSCs closely resemble the spectral signatures of hESCs. In their elegant work, metabolic differences between the two groups were hypothesized on the basis of Raman analysis. Our study offers a further, more detailed description of the biochemical diversity between hiPSCs and hESCs, thereby providing additional insights into the molecular characteristics of reprogrammed and naïve stem cells.

## Conclusions

Overall, our work confirms the usefulness of Raman spectroscopy for achieving a molecular fingerprint in hPSC discrimination, adding a label-free, optical technique to the available biochemical tools. We believe that the experimental method described in this study may contribute to increase the level of sensitivity offered nowadays by more conventional approaches. Furthermore, it may improve the capability to assess the true potential owned by hiPSCs for regenerative medicine, drug screening, and disease modeling, thus complementing other well-known in-vitro and in-vivo approaches.

## Additional file

Additional file 1: Figure S1. Showing generation and characterization of hiPSCs from skin fibroblasts. (**A**) Representative images of generated iPSCs showing ESC-like morphology and AP activity. (**B**) RT-PCR analysis showing loss of Sendai viral transgenes in hiPSCs (lane 1), their presence in infected parental fibroblasts (ipF, lane 2), and their absence in noninfected parental fibroblasts (pF, lane 3). (**C**) qRT-PCR analysis confirming upregulation of the endogenous pluripotency genes *OCT4*, *SOX2*, *c-MYC*, *REX1*, and *NANOG* in generated hiPSCs and hESCs, which are used as a control cell line. All expression values normalized to *GAPDH* and relative to the parental fibroblasts. Data represent mean ± SD. (**D**) Immunostaining of hESCs and iPSCs for pluripotency markers Oct4 (red) and Nanog (green), with costaining with DAPI (blue). Scale bar = 50 μm. (**E**) Genome-wide gene expression profiling by PluriTest of undifferentiated hESCs and iPSCs reveals that the lines have a high pluripotency score. (**F**) Immunostaining of whole EBs (day 25) for Nestin (ectoderm), Brachyury (mesoderm), and Sox17 (endoderm) and costaining with DAPI (blue). Scale bar = 50 μm. (**G**) qRT-PCR analysis showing potential of generated hiPSCs to differentiate into cells of all three germ layers (*SOX7*, endodermal marker; *HAND1*, *ACTA2*, and *MYL2*, mesodermal markers; *NESTIN* and *BMP4*, ectodermal markers) and downregulation of pluripotency-associated genes (*OCT4* and *NANOG*). All expression values normalized to *GAPDH* and relative to the respective hiPSC clones (TIF 5709 kb)

## Abbreviations

AP: Alkaline phosphatase
BSA: Bovine serum albumin
CFSE: 5,6-Carboxyfluorescein diacetate succinimidyl ester
DAPI: 4′,6-Diamidino-2-phenylindole dihydrochloride
DMEM: Dulbecco’s modified Eagle’s medium
EB: Embryoid body
FBS: Fetal bovine serum
hESC: Human embryonic stem cell
hiPSC: Human induced pluripotent stem cell
hPSC: Human pluripotent stem cell
KCA: K-means cluster analysis
M-Fish: Multiplex-fluorescence in-situ hybridization
PCA: Principal component analysis
PCR: Polymerase chain reaction
PFA: Paraformaldehyde
PI: Propidium iodide
qRT-PCR: Real-time quantitative PCR
RT-PCR: Reverse-transcription PCR
SSC: Saline–sodium citrate
