# Supplementary figures and images for: Two sides of the same coin? Unraveling subtle differences between human embryonic and induced pluripotent stem cells by Raman spectroscopy
Source: Stem Cell Res Ther. 2017 Nov 28;8:271. doi: 10.1186/s13287-017-0720-1 (PMC5706396; doi:10.1186/s13287-017-0720-1)

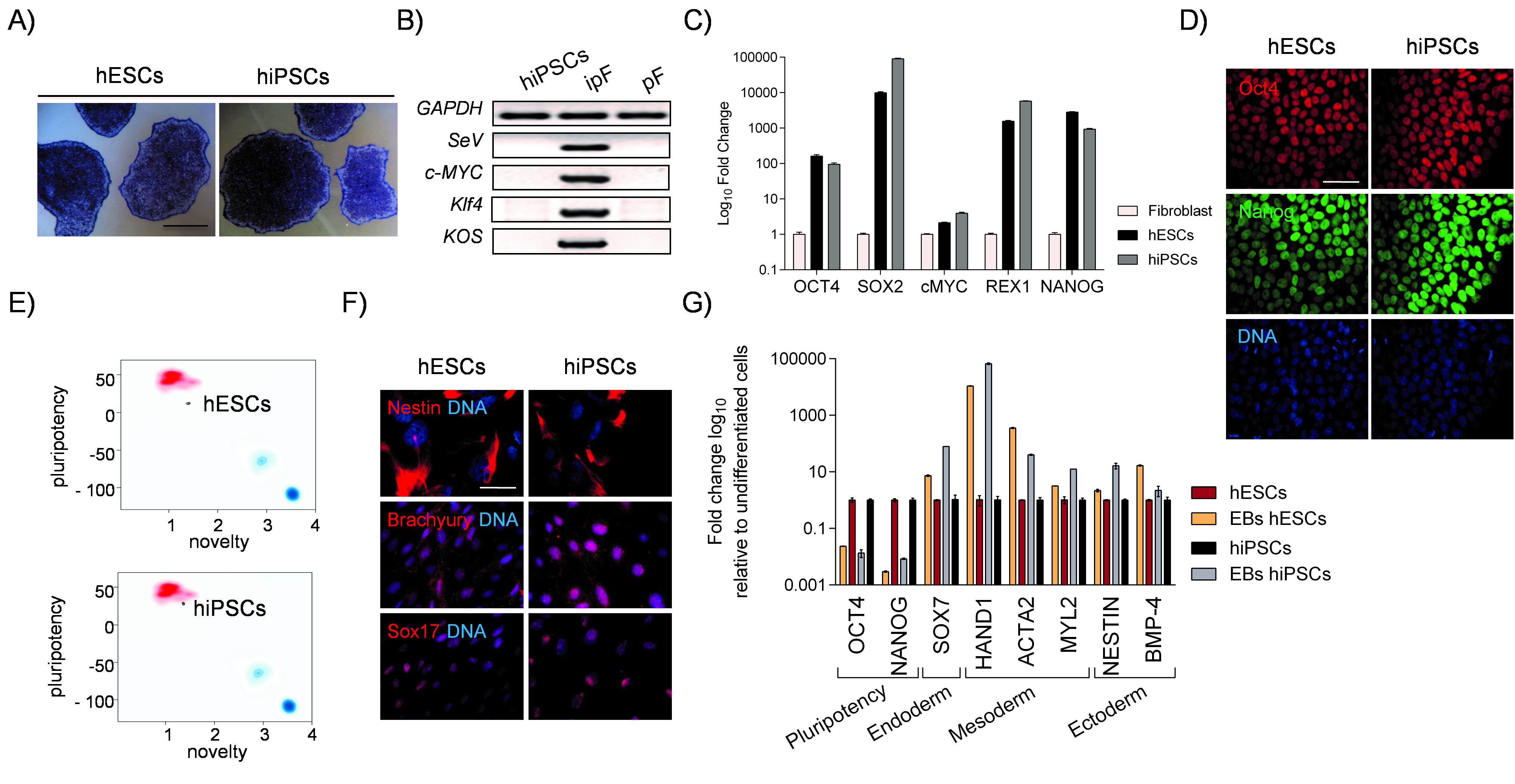

Supplement: Additional file 1: Figure S1. — Showing generation and characterization of hiPSCs from skin fibroblasts. (A) Representative images of generated iPSCs showing ESC-like morphology and AP activity. (B) RT-PCR analysis showing loss of Sendai viral transgenes in hiPSCs (lane 1), their presence in infected parental fibroblasts (ipF, lane 2), and their absence in noninfected parental fibroblasts (pF, lane 3). (C) qRT-PCR analysis confirming upregulation of the endogenous pluripotency genes OCT4, SOX2, c-MYC, REX1, and NANOG in generated hiPSCs and hESCs, which are used as a control cell line. All expression values normalized to GAPDH and relative to the parental fibroblasts. Data represent mean ± SD. (D) Immunostaining of hESCs and iPSCs for pluripotency markers Oct4 (red) and Nanog (green), with costaining with DAPI (blue). Scale bar = 50 μm. (E) Genome-wide gene expression profiling by PluriTest of undifferentiated hESCs and iPSCs reveals that the lines have a high pluripotency score. (F) Immunostaining of whole EBs (day 25) for Nestin (ectoderm), Brachyury (mesoderm), and Sox17 (endoderm) and costaining with DAPI (blue). Scale bar = 50 μm. (G) qRT-PCR analysis showing potential of generated hiPSCs to differentiate into cells of all three germ layers (SOX7, endodermal marker; HAND1, ACTA2, and MYL2, mesodermal markers; NESTIN and BMP4, ectodermal markers) and downregulation of pluripotency-associated genes (OCT4 and NANOG). All expression values normalized to GAPDH and relative to the respective hiPSC clones (TIF 5709 kb) [file 13287_2017_720_MOESM1_ESM.tif]
